# Interaction between Antibiotic Resistance, Resistance Genes, and Treatment Response for Urinary Tract Infections in Primary Care

**DOI:** 10.1128/JCM.00143-19

**Published:** 2019-08-26

**Authors:** Hanley J. Ho, Mei Xuan Tan, Mark I. Chen, Thean Yen Tan, Seok Hwee Koo, Agnes Y. L. Koong, Lok Pui Ng, Pei Lin Hu, Kee Tung Tan, Peter K. S. Moey, Eileen Y. L. Koh, Chia Siong Wong, David C. Lye, Ngiap Chuan Tan

**Affiliations:** a Department of Clinical Epidemiology, Office of Clinical Epidemiology, Analytics, and Knowledge, Tan Tock Seng Hospital, Singapore; b Infectious Disease Research and Training Office, National Centre for Infectious Diseases, Singapore; c Saw Swee Hock School of Public Health, National University of Singapore, Singapore; d Department of Laboratory Medicine, Changi General Hospital, Singapore; e Clinical Trials and Research Unit, Changi General Hospital, Singapore; f SingHealth Polyclinics, Singapore; g SingHealth-Duke NUS Family Medicine Academic Clinical Programme, Duke-NUS Medical School, Singapore; University of Iowa College of Medicine

**Keywords:** antibacterial agents, drug resistance, microbial, primary health care, therapeutics, urinary tract infection

## Abstract

Given increasing antimicrobial resistance, we aimed to determine antibiotic susceptibility and presence of resistance genes in uropathogens in primary care, factors associated with resistance to commonly prescribed antibiotics, and effect of treatment on early symptom resolution. We conducted a prospective study of primary care patients with urinary tract infection (UTI) symptoms and culture-confirmed UTI in Singapore from 2015 to 2016. Cohort characteristics and antimicrobial susceptibility of cultured isolates were analyzed. Among Enterobacteriaceae isolates, early symptom resolution (within 3 days) according to antibiotic prescribed and isolate susceptibility and factors associated with antibiotic resistance were evaluated. Of 695 symptomatic patients, 299 were urine culture positive; of these 299 patients, 259 (87%) were female. Escherichia coli was the most common uropathogen (76%). Enterobacteriaceae isolates (*n* = 283) were highly susceptible to amoxicillin-clavulanate (86%), nitrofurantoin (87%), and fosfomycin (98%), but >20% were resistant to ciprofloxacin and co-trimoxazole. Isolates resistant to appropriate indicator antibiotics were further tested to determine proportions positive for *bla*_CTX-M_ (14/26, 54%), plasmid-mediated *ampC* (12/24, 50%), *qnr* (7/69, 10%), and *fos* (1/6, 17%) resistance genes. A total of 67% of patients given antibiotics with susceptible isolates reported early resolution versus 45% given antibiotics with nonsusceptible isolates (*P* = 0.001) and 27% not treated (*P* = 0.018). On multivariable analysis, Indian ethnicity and diabetes mellitus were associated with amoxicillin-clavulanate resistance. Genitourinary abnormalities, UTI in the past 12 months, and hospitalization in the past 6 months were associated with ciprofloxacin and co-trimoxazole resistance. Patients given active empirical antibiotics were most likely to report early symptom resolution, but correlation with *in vitro* susceptibility was imperfect. Factors associated with resistance may guide the decision to obtain initial urine culture.

## Abstract (precis)

Given increasing antimicrobial resistance, we aimed to determine antibiotic susceptibility and presence of resistance genes in uropathogens in primary care, factors associated with resistance to commonly prescribed antibiotics, and effect of treatment on early symptom resolution. We conducted a prospective study of primary care patients with urinary tract infection (UTI) symptoms and culture-confirmed UTI in Singapore from 2015 to 2016.

## INTRODUCTION

Annually, about 150 million urinary tract infections (UTI) are diagnosed worldwide (1), and over half of adult women have at least one episode in their lifetime (2). UTI frequently presents in primary care, where treatment is usually empirical without urine culture or antibiotic susceptibility testing to guide treatment. This is appropriate given the practical necessity to treat without waiting for culture results, as long as causative uropathogens and their antimicrobial susceptibility profile remain predictable (3).

Escherichia coli is the major pathogen isolated in community-acquired UTIs (4), accounting for 75% of UTIs in primary care in Singapore (5). In a 2009 study of UTI in Singapore, 39% and 24% of E. coli isolates were resistant to co-trimoxazole and ciprofloxacin, respectively (5). However, antimicrobial resistance in community-acquired uropathogens has been increasing across many settings (6, 7) and is associated with poorer outcomes, including delayed symptom resolution, repeat medical consults, and disease progression due to ascending infection (8). Periodic reassessment of antimicrobial susceptibility patterns helps to identify groups where resistance should be suspected, determine optimal empirical antibiotics, and determine whether it remains appropriate to forgo susceptibility testing.

Our study evaluated the etiology, antibiotic susceptibility patterns, and prevalence of common hospital-associated resistance genes in uropathogens in primary care in Singapore. We assessed the effect of appropriate treatment on early symptom resolution and evaluated factors associated with resistance to commonly prescribed antibiotics in our setting.

## MATERIALS AND METHODS

### Setting.

We conducted a prospective cohort study in three public primary care clinics (polyclinics) in Singapore from 2015 to 2016. Polyclinics provide subsidized care for residents residing in urban housing estates, serving high proportions of elderly and patients requiring chronic disease care (9).

### Participants.

Adult male and female clinic patients aged 21 years and above and identified by the physician to have UTI-related symptoms were invited to participate and referred to a research assistant stationed on site. Informed consent was taken from all participants prior to entry into the study. All demographic and clinical data for the study were collected using a standardized questionnaire. This was either self-completed or administered by the research assistant, who then checked to ensure that there was no missing data. All recruited patients submitted urine samples for cultures. Those unable to give consent or provide a midstream urine sample were excluded.

We used an established standard for UTI diagnosis, i.e., detection of the uropathogen in the presence of clinical symptoms (10). Laboratory cutoffs for positive urine culture were isolation of ≥10^3^ CFU/ml of primary urinary pathogens (E. coli, *Klebsiella* sp., or Staphylococcus saprophyticus only) in pure culture or isolation of ≥10^5^ CFU/ml of these organisms as predominant growth, in accordance with the European guidelines for urinalysis (11). The significance cutoff for other potential pathogens was ≥10^5^ CFU/ml.

### Pathogen identification and antibiotic susceptibility testing.

Uropathogens were identified using conventional phenotypic methods, with antibiotic susceptibility determined by Vitek N257 cards (bioMérieux, France), with supplemental disc susceptibility testing for fosfomycin, trimethoprim, and cephalexin. Categorical susceptibility breakpoints followed current guidelines from the Clinical and Laboratory Standards Institute (12), except for Klebsiella pneumoniae susceptibility to fosfomycin, where breakpoints from a published study were used (13).

### Antibiotic resistance genotyping.

Molecular testing for selected plasmid-mediated resistance genes commonly found in the local hospital setting was performed for isolates with resistance to appropriate indicator antibiotics as follows: CTX-M extended-spectrum beta-lactamases (ESBL) and plasmid-mediated *ampC* genes for ceftriaxone-resistant isolates of E. coli and K. pneumoniae (14, 15), *qnr* genes for ciprofloxacin-resistant isolates (16), and *fos* genes for fosfomycin-resistant isolates (*fosA* being plasmid-mediated in E. coli) (17).

### Follow-up of participants.

Participants were followed up using a standardized telephone-administered questionnaire at least 4 days after their consultation and asked about resolution of urinary symptom(s), including when each symptom resolved. The study endpoint was early symptom resolution, defined as having all symptoms resolved within 3 days of initial consultation date.

### Statistical analysis.

Cohort characteristics and antimicrobial susceptibility patterns of uropathogens are described. Among patients with uropathogens from the Enterobacteriaceae family, we assessed the distribution of prescribed antibiotics among treated patients and the number of patients not treated with antibiotics (as per the clinical assessment of the attending physician). We then compared early symptom resolution outcomes according to antibiotic prescribed (if any) and the uropathogen’s susceptibility pattern. We also evaluated patient characteristics associated with early symptom resolution and factors correlated with resistance to amoxicillin-clavulanate, ciprofloxacin, co-trimoxazole, and ceftriaxone.

Pearson’s chi-square or Fisher’s exact test were used to evaluate differences in proportions for categorical variables. We calculated crude odds ratios (ORs) with 95% confidence intervals (CIs) for demographic and clinical factors associated with antibiotic resistance and early symptom resolution and adjusted ORs for multivariable logistic regression analyses using key clinical variables (with *P* values of <0.10 on univariate analysis) and adjusting for potential demographic confounders (age, gender, and ethnicity). All analyses were performed using Stata version 15 (StataCorp, College Station, TX). A *P* value of <0.05 was considered statistically significant.

## RESULTS

### Participant characteristics.

Over the 2-year study period, 743 patients with UTI-related symptoms were screened across the three study sites, of whom 695 (94%) were eligible and agreed to participate. A total of 299 had positive urine culture according to the predefined laboratory cutoffs and were included in our analyses. This comprised 259 (87%) females and 40 males (Table 1), who were generally elderly (mean age, 60.8 ± 17.3 years) and predominantly of Chinese ethnicity (*n* = 222, 74%). Substantial proportions reported physician-diagnosed UTI in the past 12 months (*n* = 115, 39%), diabetes mellitus (DM) (*n* = 76, 25%), genitourinary (GU) abnormalities (*n* = 42, 14%), antibiotic use in the past 4 weeks (*n* = 49, 16%), and hospitalization in the past 6 months (*n* = 49, 16%). A total of 270 (90%) participants completed follow-up, with 164 (55%) reporting early symptom resolution.

**TABLE 1:** Demographic and clinical factors and outcomes in male and female patients presenting with culture-positive urinary tract infection

Characteristic	Total no. (%) (*n* = 299)	No. female (%) (*n* = 259)	No. male (%) (*n* = 40)
Mean age (yr) (SD)	60.8 (17.3)	59.9 (17.6)	66.5 (14.1)
Age group (yr)			
21–29	25 (8)	23 (9)	2 (5)
30–39	17 (6)	16 (6)	1 (3)
40–49	23 (8)	23 (9)	0 (0)
50–59	61 (20)	56 (22)	5 (13)
60–69	75 (25)	62 (24)	13 (33)
70–79	63 (21)	48 (19)	15 (38)
80 and above	35 (12)	31 (12)	4 (10)
Ethnicity			
Chinese	222 (74)	198 (76)	24 (60)
Malay	31 (10)	26 (10)	5 (13)
Indian	29 (10)	20 (8)	9 (23)
Others	17 (6)	15 (6)	2 (5)
Medical history			
Physician-diagnosed UTI in past 12 mo	115 (39)	102 (39)	13 (33)
Hospitalization within the last 6 mo	37 (12)	28 (11)	9 (23)
Diabetes mellitus	76 (25)	59 (23)	17 (43)
Genitourinary abnormalities	42 (14)	32 (12)	10 (25)
Use of antibiotics within last 4 wks	49 (16)	43 (17)	6 (15)
Outcomes during telephone follow-up			
Successfully contacted for follow-up	270 (90)	235 (91)	35 (88)
Symptoms resolved within 3 days	164 (55)	151 (58)	13 (33)

### Distribution of uropathogens and antibiotic susceptibility patterns.

We detected 266 isolates from females and 40 from males. Overall, the most common pathogen was Escherichia coli (*n* = 231, 76%), followed by Klebsiella pneumoniae (*n* = 20, 7%) and Proteus mirabilis (*n* = 14, 5%). A further 18 (6%) isolates were other Enterobacteriaceae, with remaining isolates being Streptococcus agalactiae (*n* = 8, 3%), Enterococcus faecalis (*n* = 6, 2%), and Staphylococcus saprophyticus (*n* = 3, 1%). This overall distribution was similar for females. Among males, E. coli was the most common pathogen detected (*n* = 25, 63%), with small numbers of other isolates including E. faecalis (*n* = 3, 8%), K. pneumoniae (*n* = 2, 5%), and Citrobacter koseri (*n* = 2, 5%).

Among Enterobacteriaceae (Table 2), high proportions of isolates were susceptible to amoxicillin-clavulanate (86%), ceftibuten (91%), nitrofurantoin (87%), and fosfomycin (98%). However, <80% were susceptible to ciprofloxacin (76%), levofloxacin (76%), and co-trimoxazole (74%). Isolates also demonstrated high susceptibility to parenterally administered antibiotics (minimum of 89% for gentamicin). Using only data from female participants gave similar results (see Table S1 in the supplemental material), but numbers were insufficient for a corresponding assessment in males.

**TABLE 2:** Antibiotic susceptibility profiles of Enterobacteriaceae isolated from urine cultures (*n* = 283)

Antibiotic	Escherichia coli (*n* = 231)			Other *Enterobacteriaceae*^*a*^ (*n* = 52)		
	Total no. tested	No. susceptible	%	Total no. tested	No. susceptible	%
Oral						
Amoxicillin	231	114	49	52	13	25
Amoxicillin-clavulanate	231	206	89	52	37	71
Ceftibuten	87	77	89	21	21	100
Cefuroxime	231	173	75	52	41	79
Cephalexin	231	60	26	52	36	69
Ciprofloxacin	231	165	71	52	50	96
Co-trimoxazole	231	164	71	52	46	89
Fosfomycin	231	227	98	20	19	95
Levofloxacin	143	102	71	31	30	97
Nitrofurantoin	230	228	99	52	17	33
Trimethoprim	231	161	70	52	44	85
Parenteral						
Amikacin	231	231	100	52	52	100
Aztreonam	231	206	89	52	51	98
Cefepime	231	213	92	52	52	100
Cefotaxime	143	127	89	31	30	97
Cefoxitin	231	218	94	52	39	75
Ceftazidime	231	205	89	52	51	98
Ceftriaxone	231	205	89	52	51	98
Ertapenem	231	231	100	52	51	98
Gentamicin	231	201	87	52	50	96
Imipenem	231	231	100	51	35	69
Meropenem	231	231	100	52	52	100
Piperacillin-tazobactam	231	218	94	51	50	98

a Includes Klebsiella pneumoniae (20), Proteus mirabilis (14), Enterobacter aerogenes (7), Citrobacter koseri (6), Serratia marcescens (2), Citrobacter werkmanii (1), Enterobacter cloacae (1), and Morganella morganii (1).

**FIG 1:**
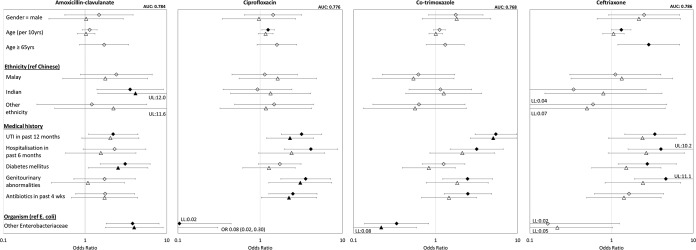
Univariate and multivariable analysis of factors associated with resistance to various antibiotics for urinary tract infections caused by Enterobacteriaceae. Diamonds indicate univariate odds ratios, and triangles indicate multivariable odds ratios. Error bars indicate 95% confidence intervals. Shaded diamonds and triangles denote statistically significant variables. UTI, urinary tract infection; LL, lower limit; UL, upper limit; AUC, area under the curve.

Figure 1 shows factors associated with resistance. Amoxicillin-clavulanate resistance was positively associated with Indian ethnicity (versus Chinese), physician-diagnosed UTI in the past 12 months, hospitalization in the past 6 months, and DM. Ciprofloxacin, co-trimoxazole, and ceftriaxone resistance were positively associated with recent UTI, hospitalization, or GU abnormalities (all three); age group (ciprofloxacin and ceftriaxone); use of antibiotics in the past 4 weeks (ciprofloxacin and co-trimoxazole); and history of DM (ceftriaxone only).

On multivariable analysis, factors independently associated with amoxicillin-clavulanate resistance were Indian ethnicity (adjusted odds ratio [AOR] = 4.14; 95% CI, 1.43 to 11.99; *P* = 0.01) and DM (AOR = 2.54; 95% CI, 1.09 to 5.88; *P* = 0.03). Factors independently associated with ciprofloxacin, co-trimoxazole, and ceftriaxone resistance were recent UTI (all three) as well as GU abnormalities and recent hospitalization (for ciprofloxacin only).

### Antibiotic treatment, resistance, and early symptom resolution.

Of 278 participants with Enterobacteriaceae isolates, 265 (95%) were given empirical antibiotics during the initial consultation, most commonly amoxicillin-clavulanate (63%), ciprofloxacin (26%), and co-trimoxazole (6%). A total of 83% of antibiotics prescribed were active against the isolates for that patient as follows: 88% for nitrofurantoin and 87% for amoxicillin-clavulanate but only 76% for ciprofloxacin and 69% for co-trimoxazole (*P* value across treatment groups = 0.047).

**FIG 2:**
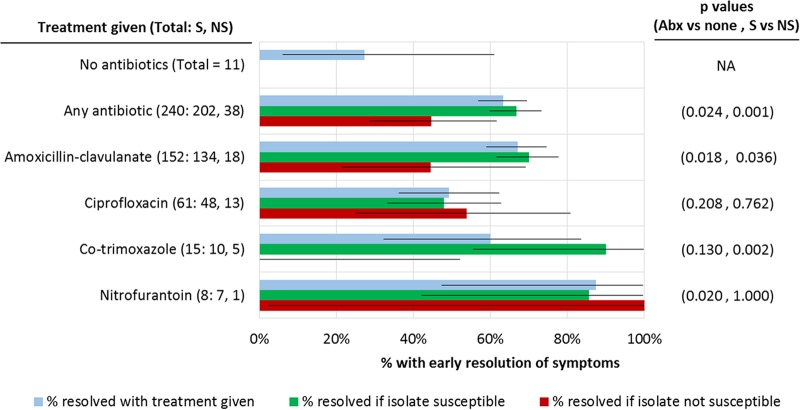
Early symptom resolution by antibiotic given and antibiotic susceptibility. Horizontal lines in bar graph indicate 95% confidence intervals for proportion resolved with treatment given. (Left) Total, total number of patients given that treatment; S, treated patients with susceptible isolates; NS, treated patients with nonsusceptible isolates; Abx, antibiotic. (Right) *P* values compare proportions resolved in patients given that treatment versus patients given no antibiotics (Abx vs none) and in treated patients with susceptible isolates versus treated patients with nonsusceptible isolates (S vs NS).

Among patients with follow-up data (Fig. 2), 152/240 (63%) treated with any empirical antibiotics at initial consultation reported early symptom resolution, which was significantly higher than 3/11 (27%) patients not given empirical antibiotics (*P* = 0.024). Within the treated group, 135/202 (67%) patients with susceptible isolates reported early resolution versus 17/38 (45%) with nonsusceptible isolates (*P* = 0.001). Corresponding proportions for those prescribed amoxicillin-clavulanate were 94/134 (70%) versus 8/18 (44%) (*P* = 0.036); for co-trimoxazole, proportions were 9/10 (90%) versus 0/5 (0%) (*P* = 0.002); and for nitrofurantoin, proportions were 7/8 (88%) versus 1/1 (100%) (*P* = 1.000). For ciprofloxacin, however, only 23/48 (48%) treated patients with susceptible isolates reported early resolution versus 7/13 (54%) treated patients with nonsusceptible isolates (*P* = 0.762).

We further evaluated possible reasons for the observed outcomes with ciprofloxacin. Table S2 in the supplemental material shows that patient characteristics across different antibiotic treatment groups did not have major differences, except that the proportion of patients with DM varied across patients treated with ciprofloxacin (36%), co-trimoxazole (40%), nitrofurantoin (50%), and amoxicillin-clavulanate (20%). On stratification by dosage of ciprofloxacin prescribed (250 mg twice a day versus 500 mg twice a day), 5/16 (31%) patients given 250 mg twice a day reported early resolution versus 25/45 (56%) patients given 500 mg twice a day (*P* = 0.095).

On multivariable analysis of factors associated with early symptom resolution (Table 3), male patients were significantly less likely to report resolution. Compared to no antibiotic treatment, treatment of nonsusceptible isolates with any antibiotic was not significantly associated with symptom resolution. Treatment of susceptible isolates with amoxicillin-clavulanate or “other antibiotics” (co-trimoxazole or nitrofurantoin) was significantly associated with resolution, but treatment with ciprofloxacin remained nonsignificant.

**TABLE 3:** Univariate and multivariable analyses of factors associated with early resolution of symptoms

Factor	Univariate			Multivariable		
	OR	95% CI	*P* value	AOR	95% CI	*P* value
Gender, male	0.34	0.15–0.76	0.008	0.34	0.14–0.83	0.018
Age, per 10-yr increase	0.96	0.82–1.11	0.563	1.03	0.86–1.24	0.745
Ethnicity (vs Chinese)						
Malay	0.70	0.30–1.59	0.39	0.75	0.29–1.93	0.547
Indian	0.75	0.33–1.69	0.481	1.09	0.42–2.86	0.859
Others	1.19	0.35–4.11	0.780	1.44	0.36–5.65	0.605
Medical history						
Physician-diagnosed UTI in past 12 mo	0.66	0.39–1.11	0.116			
Hospitalization within the last 6 mo	0.83	0.37–1.84	0.646			
Diabetes mellitus	0.55	0.31–0.98	0.042	0.77	0.38–1.53	0.448
Genitourinary abnormalities	0.51	0.25–1.06	0.073	0.69	0.30–1.58	0.385
Use of antibiotics within last 4 wks	0.72	0.37–1.39	0.324			
Antibiotic given and susceptibility (vs no antibiotic)						
Given amoxicillin-clavulanate, not susceptible	2.13	0.42–10.78	0.359	2.27	0.43–12.10	0.337
Given ciprofloxacin, not susceptible	3.11	0.56–17.33	0.195	2.94	0.50–17.16	0.232
Given other antibiotic, not susceptible	0.53	0.04–6.65	0.625	0.62	0.05–8.51	0.723
Given amoxicillin-clavulanate, susceptible	6.27	1.58–24.85	0.009	6.11	1.49–25.12	0.012
Given ciprofloxacin, susceptible	2.45	0.58–10.38	0.223	2.38	0.54–10.39	0.250
Given other antibiotic, susceptible	20.00	2.75–145.48	0.003	22.37	2.89–173.19	0.003

### Prevalence of resistance genes and early symptom resolution.

A total of 14/26 (54%) E. coli and K. pneumoniae isolates with reduced susceptibility to ceftriaxone were positive for *bla*_CTX-M_ alleles. A total of 12/24 (50%) E. coli and K. pneumoniae isolates screened for *ampC* genes were positive. Four E. coli isolates were positive for both *ampC* and *bla*_CTX-M_ genes, while another 4 had neither cephalosporinase detected. *qnr* resistance genes were detected in 7 (10%) of 69 isolates with reduced quinolone susceptibility. Finally, only 1/6 (17%) tested positive for *fos* genes (*fosA96*). In total, 28 isolates were positive for at least one of these resistance genes (common in the local hospital setting), but only 7 (25%) belonged to patients reporting hospitalization in the past 6 months.

Among patients treated using amoxicillin-clavulanate, 3/3 (100%) patients with isolates negative for *bla*_CTX-M_ and *ampC* genes reported early resolution versus 3/5 (60%) with *bla*_CTX-M_ only, 2/5 (40%) with *ampC* only, and 0/3 (0%) when both *ampC* and *bla*_CTX-M_ were detected (*P* = 0.091).

## DISCUSSION

In our study of a predominantly female cohort, with a high proportion of elderly, we observed high levels of resistance to ciprofloxacin and co-trimoxazole, which were still being prescribed. For amoxicillin-clavulanate, which was most commonly prescribed, those with resistant isolates were less likely to report early symptom resolution. Moreover, genotypic markers of plasmid-mediated resistance were identified in a substantial number of isolates.

When compared with proportions of E. coli isolates from the study in 2009 (5), similar proportions were susceptible to amoxicillin-clavulanate (89% versus 91% in 2009) and ciprofloxacin (71% versus 76%), but the proportion susceptible to co-trimoxazole was slightly higher (71% versus 62%; *P* = 0.049). However, the 2009 study relied on retrospective chart reviews of urine cultures. Since physicians may selectively test more complicated patients or those who fail treatment (18), that study may have overestimated antibiotic resistance levels compared to those in our current study, which prospectively obtained urine cultures from all UTI patients and arguably provides a more accurate assessment of current antibiotic susceptibility profiles.

Globally, antibiotic susceptibility of uropathogens varies widely (19). We observed less resistance of E. coli to amoxicillin-clavulanate compared to that of some other community settings (7, 20). Ciprofloxacin and co-trimoxazole resistance was comparable to that of data from primary care in Hong Kong (21) and less prevalent than in India, Iran, and Turkey (20). Resistance in uncomplicated UTIs may be correlated with antibiotic consumption (22). Antibiotics cannot be obtained without prescription by licensed physicians in Singapore. However, one study suggests large variations in primary care prescriptions of antibiotics for upper respiratory tract infection (from 0% to 70% of episodes) (23). The moderate levels of resistance observed in Singapore should hence motivate additional legislative (24) and physician education interventions to improve antibiotic stewardship (25).

Among Enterobacteriaceae, factors independently associated with resistance to amoxicillin-clavulanate were Indian ethnicity and DM. Potential explanations may lie in travel-related exposures to the high prevalence of antibiotic-resistant bacteria in South Asia for the former (26) and greater exposure of DM patients to health care interventions for the latter (27). Associations between ciprofloxacin and co-trimoxazole resistance with past UTI diagnosis is consistent with extant literature (28, 29), and the association between ciprofloxacin resistance and hospitalization may be related to the substantially higher resistance prevalence in hospital settings (30).

Regardless of the specific patterns of resistance, we believe that the findings with wider relevance were our follow-up data on how matching empirical antibiotic to uropathogen susceptibility affects early symptom resolution, but also how this relationship was imperfect. The small proportion (27%) reporting early resolution despite not receiving empirical antibiotics was consistent with the clinical course of untreated uncomplicated cystitis, where 25% to 42% may experience resolution (4). In those given antibiotics, the “90/60” rule has been proposed, where infections due to susceptible and resistant isolates would correspondingly respond 90% and 60% of the time (31). In our study, 67% of treated patients with susceptible isolates and 45% of treated patients with nonsusceptible isolates reported early resolution. Our short follow-up duration may account for the lower proportions reporting symptom resolution (67/45 instead of 90/60), though interestingly, the ratio between groups appeared to be consistent with the 90/60 rule.

However, while we demonstrated the overall impact of appropriate empirical antibiotic therapy on early symptom resolution, our findings also caution us about the validity of extrapolating efficacy purely based on *in vitro* susceptibility testing alone. In patients prescribed amoxicillin-clavulanate, symptoms appeared less likely to resolve when genotypic resistance markers were present than when they were absent. For those prescribed ciprofloxacin, early symptom resolution rates were lowest (about 50%), and outcomes did not differ significantly between patients with susceptible and nonsusceptible isolates. There were no striking differences in patients prescribed ciprofloxacin compared to those prescribed other antibiotics to account for these intriguing results. However, underdosing may have contributed to the poorer than expected response. Patients prescribed a dose of 250 mg twice a day appeared to have poorer outcomes than those given 500 mg twice a day, although this observation was likely underpowered (*P* = 0.095). These doses were chosen by primary care doctors and might not have represented any renal dose adjustment or adherence to professional guidelines.

The effectiveness of ciprofloxacin is a function of peak concentration divided by MIC (32). Particularly in the elderly, where there is decreased renal excretion, the drug may not reach sufficient concentrations in the urine with a 250-mg twice daily prescription. A 500-mg once a day dosing regimen has been shown to deliver higher drug concentrations in the urine, and this regimen at minimum should be prescribed to optimize outcomes (32).

Overall, with regard to empirical therapy, the vast majority of isolates remained susceptible to fosfomycin and nitrofurantoin. Both of these are recommended antibiotics for uncomplicated UTIs in international guidelines (over amoxicillin-clavulanate, which is thought to have lower efficacy, more adverse effects, and broader spectrum of cover) (19) and are viable alternatives that should be considered for Singapore. Given the levels of resistance observed (>20%), ciprofloxacin and co-trimoxazole are less preferred in our setting without ordering urine cultures to guide treatment. In turn, the need for urine cultures could be guided by the risk factors highlighted in this study. Furthermore, appropriate dosing of ciprofloxacin, as discussed above, is vital to ensuring a favorable response to therapy.

Only a low proportion of isolates (<10%) in our primary care setting carried the *bla*_CTX-M_, *ampC*, and *qnr* resistance genes, which are highly prevalent among hospital Enterobacteriaceae isolates in Singapore (33, 34). However, of these, 75% were not associated with hospitalization in the past 6 months, and acquisition may thus be occurring within the community. The rising prevalence of resistance, including strains carrying key resistance genes with possible clinical significance, compounded by the imperfect correlation between susceptibility testing results with actual clinical effectiveness, is likely an emerging and important issue which will increasingly complicate management of UTI in primary care settings around the world.

In the face of rapidly changing resistance profiles, strategies are urgently needed for rationalizing how best to determine guidelines for UTI treatment outside of the hospital, where there is typically no routine surveillance or follow up with patients. We argue that a robust approach may need to go beyond surveillance of antimicrobial susceptibility testing results and include routine monitoring of a sample of patients for symptom resolution and possibly the use of genotypic markers of resistance.

The strengths of our study include using a prospective (rather than a potentially biased retrospective) approach to determine the distribution of uropathogens presenting in the primary care setting and their antibiotic susceptibility profiles, with the cohort design demonstrating how antibiotic choices potentially interact with susceptibility profiles and genotypic markers of resistance to affect a clinically relevant endpoint. However, our study has some limitations. Our cohort was predominantly elderly and reflective of the catchment of polyclinics, which provide subsidized primary care services, though this may also reflect the ageing of the population in Singapore (35). We did not split and analyze patients according to different types of UTI (from uncomplicated cystitis to acute pyelonephritis). However, our intent in this paper was to provide generalizable results for UTI in primary care. Some culture results obtained may have been false positives, which would have affected the accuracy of our study, although this number is likely to be small, since the criteria for recruitment were initial presentation with symptoms of UTI. Our symptom resolution endpoint may have been too brief to document complications and risk of recurrent UTI known to occur with inappropriate treatment (8), and we had only a small number of patients untreated with antibiotics for comparison. Some subgroup analyses were also limited by the sample size. Our study only focused on patients with culture-positive UTIs, although a recent study showed that a high proportion of symptomatic women with negative urine cultures did actually have UTI, most commonly due to E. coli (36). Further studies are needed to evaluate whether treatment outcomes in culture-negative patients are similar to culture-positive patients.

In conclusion, our study profiled antibiotic susceptibilities for uropathogens in our primary care setting and identified risk factors for resistance, which may guide the decision to obtain initial urine culture to guide therapy. While patients given active empirical antibiotics were most likely to report early symptom resolution, the correlation with *in vitro* susceptibility was imperfect. Strategies are needed to monitor clinical effectiveness of treatment options for UTI in primary care in the face of rapidly changing resistance patterns.

## Supplementary Material

Supplemental file 1
