# Supplementary material for: Interaction between Antibiotic Resistance, Resistance Genes, and Treatment Response for Urinary Tract Infections in Primary Care
Source: J Clin Microbiol. 2019 Aug 26;57(9):e00143-19. doi: 10.1128/JCM.00143-19 (PMC6711900; doi:10.1128/JCM.00143-19)
Supplement: Supplemental file 1 [file JCM.00143-19-s0001.pdf]

	Escherichia coli (n=202)			Other Enterobacteriaceae* (n=42)		
	**Total tested**	**Susceptible**	**(%)**	**Total tested**	**Susceptible**	**(%)**
Antibiotics - Oral						
Amoxicillin	202	103	(51)	42	12	(29)
Amoxicillin-clavulanate	202	182	(90)	42	31	(74)
Ceftibuten	79	71	(90)	19	19	(100)
Cefuroxime	202	156	(77)	42	34	(81)
Cephalexin	202	57	(28)	42	32	(76)
Ciprofloxacin	202	147	(73)	42	40	(95)
Co-trimoxazole	202	147	(73)	42	37	(88)
Fosfomycin	202	199	(99)	17	16	(94)
Levofloxacin	122	86	(71)	23	22	(96)
Nitrofurantoin	201	199	(99)	42	15	(36)
Trimethoprim	202	144	(71)	42	36	(86)
Antibiotics - Parenteral						
Amikacin	202	202	(100)	42	42	(100)
Aztreonam	202	184	(91)	42	41	(98)
Cefepime	202	188	(93)	42	42	(100)
Cefotaxime	122	111	(91)	23	22	(96)
Cefoxitin	202	193	(96)	42	34	(81)
Ceftazidime	202	183	(91)	42	41	(98)
Ceftriaxone	202	183	(91)	42	41	(98)
Ertapenem	202	202	(100)	42	41	(98)
Gentamicin	202	176	(87)	42	40	(95)
Imipenem	202	202	(100)	42	28	(67)
Meropenem	202	202	(100)	42	42	(100)
Piperacillin-tazobactam	202	193	(96)	42	41	(98)

Table S1. Antibiotic susceptibility profiles of Enterobacteriaceae isolated from urine cultures of female patients only (n=244).*Includes *Klebsiella pneumoniae* (17), *Proteus mirabilis* (13), *Enterobacter aerogenes* (6), *Citrobacter koseri* (4), *Citrobacter werkmanii* (1), and *Morganella morganii* (1).

	Total (N=236) N	(%)	Amoxicillin-clavulanate (N=152) N	(%)	Ciprofloxacin (N=61) N	(%)	Co-trimoxazole (N=15) N	(%)	Nitrofurantoin (N=8) N	(%)	p value
Gender = male	27	(11%)	17	(11%)	7	(12%)	2	(13%)	1	(13%)	0.977
Age group (yrs)											
20 - 29	21	(9%)	16	(11%)	3	(5%)	2	(13%)	0	(0%)	0.358
30 - 39	15	(6%)	11	(7%)	3	(5%)	1	(7%)	0	(0%)	
40 - 49	18	(8%)	12	(8%)	4	(7%)	1	(7%)	1	(13%)	
50 - 59	52	(22%)	36	(24%)	10	(16%)	4	(27%)	2	(25%)	
60 - 69	59	(25%)	36	(24%)	21	(34%)	1	(7%)	1	(13%)	
70 - 79	46	(20%)	28	(18%)	12	(20%)	2	(13%)	4	(50%)	
80 and above	25	(11%)	13	(9%)	8	(13%)	4	(27%)	0	(0%)	
Ethnicity											
Chinese	174	(74%)	114	(75%)	46	(75%)	10	(67%)	4	(50%)	0.050
Malay	25	(11%)	17	(11%)	4	(7%)	1	(7%)	3	(38%)	
Indian	26	(11%)	11	(7%)	10	(16%)	4	(27%)	1	(13%)	
Others	11	(5%)	10	(7%)	1	(2%)	0	(0%)	0	(0%)	
Medical history											
Physician-diagnosed UTI in past 12 months	92	(39%)	60	(40%)	21	(34%)	7	(47%)	4	(50%)	0.710
Hospitalisation within the last 6 months	27	(11%)	18	(12%)	4	(7%)	3	(20%)	2	(25%)	0.166
Diabetes mellitus	63	(27%)	31	(20%)	22	(36%)	6	(40%)	4	(50%)	0.017
Genitourinary abnormalities	32	(14%)	22	(15%)	6	(10%)	2	(13%)	2	(25%)	0.527
Use of antibiotics within last 4 weeks	41	(17%)	22	(15%)	16	(26%)	3	(20%)	0	(0%)	0.128

Table S2. Comparison of patient characteristics by antibiotic prescribed, among patients with follow-up data. P values are based on Fisher's Exact test. UTI = urinary tract infection.
